# Case Report: Novel *ADAMTSL2* compound heterozygous mutations in geleophysic dysplasia with bilateral glaucoma and keratoconus-like corneal ectasia

**DOI:** 10.3389/fgene.2026.1751809

**Published:** 2026-01-26

**Authors:** Chung-Lin Lee, Chih-Kuang Chuang, Huei-Ching Chiu, Ya-Hui Chang, Yuan-Rong Tu, Yun-Ting Lo, Jun-Yi Wu, Hsiang-Yu Lin, Shuan-Pei Lin

**Affiliations:** 1 Department of Pediatrics, MacKay Memorial Hospital, Taipei, Taiwan; 2 Institute of Clinical Medicine, National Yang-Ming Chiao-Tung University, Taipei, Taiwan; 3 International Rare Disease Center, MacKay Memorial Hospital, Taipei, Taiwan; 4 Department of Medicine, Mackay Medical University, New Taipei City, Taiwan; 5 Mackay Junior College of Medicine, Nursing and Management, Taipei, Taiwan; 6 Division of Genetics and Metabolism, Department of Medical Research, MacKay Memorial Hospital, Taipei, Taiwan; 7 College of Medicine, Fu-Jen Catholic University, Taipei, Taiwan; 8 Department of Medical Research, China Medical University Hospital, China Medical University, Taichung, Taiwan; 9 Department of Infant and Child Care, National Taipei University of Nursing and Health Sciences, Taipei, Taiwan

**Keywords:** ADAMTSL2 gene, geleophysic dysplasia, glaucoma, joint contractures, keratoconus-like corneal ectasia, skeletal dysplasia

## Abstract

Geleophysic dysplasia represents an exceedingly uncommon autosomal recessive skeletal disorder marked by profound growth restriction, contractures affecting multiple joints, and cardiac valve abnormalities. The molecular foundation involves *ADAMTSL2* gene mutations disrupting extracellular matrix architecture. We document a 29-year-old Taiwanese woman followed longitudinally for 25 years, presenting with severe short stature measuring 141.2 cm, widespread joint contractures, thoracolumbar scoliosis, and distinctive gait abnormalities. Whole-exome sequencing identified compound heterozygous *ADAMTSL2* mutations: c.286C>T resulting in p. Arg96Trp and c.454_459del causing p. Cys152_Thr153del deletion. The clinical course revealed musculoskeletal deterioration alongside mild mitral valve involvement and os odontoideum. Bilateral glaucoma, consistent with previously reported ocular manifestations in geleophysic dysplasia, was diagnosed at age 26. Notably, recent ophthalmologic evaluation revealed keratoconus-like corneal ectasia with paradoxically increased central corneal thickness measuring 690–693 μm bilaterally, substantially exceeding normal values of 520–560 μm. This paradoxical corneal thickening, contrasting with the stromal thinning characteristic of classical keratoconus, represents a novel *ADAMTSL2*-related corneal phenotype. The patient maintained normal intellectual capacity despite physical limitations, contrasting with published early mortality rates approaching 33%. This extended clinical documentation establishes keratoconus-like corneal ectasia with paradoxical corneal thickening as a novel ophthalmologic manifestation in geleophysic dysplasia, while adding to prior reports of glaucoma in this condition. These findings emphasize the necessity for comprehensive ophthalmologic monitoring in *ADAMTSL2*-related disorders and supporting multidisciplinary management strategies.

## Introduction

Geleophysic dysplasia constitutes an extraordinarily rare autosomal recessive skeletal condition characterized by marked short stature, progressive contractures involving multiple joints, recognizable facial characteristics, and potentially fatal cardiac complications (Le Goff et al., 2008). The terminology originates from Greek words describing the characteristic pleasant facial expression typically observed in affected individuals (Spranger et al., 1984). Global medical literature documents fewer than 100 cases, with estimated prevalence approximating one per million individuals (Bonafe et al., 2015).

Clinical manifestations encompass diverse progressive multisystem features typically emerging during early childhood (Marzin et al., 2021). Affected individuals uniformly display severe growth restriction, with adult heights representing one to three standard deviations below population norms (Camarena et al., 2024). Skeletal abnormalities accompany progressive contractures affecting both large and small joints, with the characteristic tip-toe gait observed in virtually all patients (Morales et al., 2025). Contemporary whole-exome sequencing approaches have substantially enhanced diagnostic accuracy, achieving detection rates between 65 and 74 percent for suspected skeletal dysplasia cases (Han et al., 2020).

Beyond musculoskeletal involvement, cardiac valvular disease occurs in approximately 68 percent of cases and primarily determines prognosis (Faivre et al., 2003). The molecular basis centers on *ADAMTSL2* mutations encoding a 951-amino acid secreted glycoprotein essential for extracellular matrix organization and transforming growth factor-beta bioavailability regulation (Le Goff and Cormier-Daire, 2011). Loss of *ADAMTSL2* function produces dysregulated extracellular matrix architecture through impaired microfibril assembly and composition, particularly affecting fibrillin-1 and fibrillin-2 interactions. While transforming growth factor-beta dysregulation has been observed in select patient contexts, recent evidence indicates that transforming growth factor-beta elevation is not a universal feature of *ADAMTSL2*-related disease (Camarena et al., 2024). The primary pathogenic mechanism involves direct disruption of fibrillin microfibril assembly and composition, independent of transforming growth factor-beta signaling. This dysregulation leads to disrupted microfibril assembly affecting skeletal growth and tendon function, progressive cardiac valve fibrosis, airway dysplasia with glycoprotein storage accumulation, and aberrant cellular migration patterns within connective tissues (Hubmacher et al., 2011; Camarena et al., 2024).

Current understanding has evolved substantially with recognition of genetic heterogeneity within the acromelic dysplasia spectrum. Recent genotype-phenotype correlation studies demonstrate that clinical severity correlates directly with the degree of impaired *ADAMTSL2* protein secretion (Allali et al., 2011). Despite advances in molecular understanding, the extreme rarity of the condition, combined with its progressive nature and high early mortality rate of approximately 33 percent before age 5 years, necessitates continued documentation of individual cases to better understand the clinical spectrum (Cheng et al., 2018).

## Case presentation

### Patient demographics and background

**FIGURE 1:**
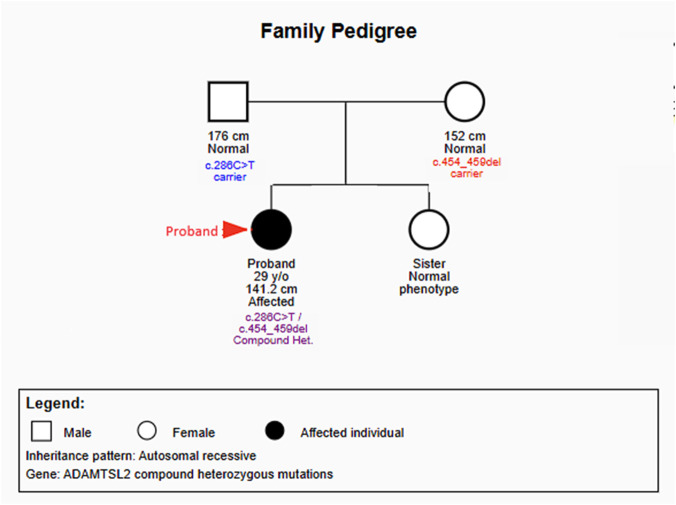
Family pedigree showing autosomal recessive inheritance pattern. The proband (indicated by arrow) carries compound heterozygous *ADAMTSL2* mutations: c.286C>T (inherited from father) and c.454_459del (inherited from mother). Both parents are asymptomatic carriers. Squares represent male family members; circles represent female family members; filled symbols indicate affected individuals.

We present a 29-year-old Taiwanese woman with comprehensive 25-year clinical follow-up of progressive skeletal dysplasia. She was born at full term with birth weight 2,770 g to nonconsanguineous parents. Family history revealed paternal height 176 cm and maternal height 152 cm, with her younger sister displaying normal phenotype. No other family members exhibited skeletal dysplasia or similar clinical features (Figure 1).

During early childhood, the patient achieved normal developmental milestones and demonstrated above-average academic performance. Her intellectual development remained unimpaired despite progressive physical manifestations.

### Clinical history and evolution

The patient’s clinical course demonstrated progressive functional deterioration across multiple organ systems throughout the 25-year follow-up period. She displayed persistent short stature with current measurements 141.2 cm height, 47 kg weight, and 23.6 kg per square meter body mass index. Longitudinal growth tracking revealed stable height at 143 cm from ages 16–28 years, followed by reduction to 141.2 cm at age 29 years. This measurement represents approximately four to five standard deviations below population means for adult women. This 1.8 cm height reduction, confirmed on multiple measurements in 2025, most likely reflects progressive thoracolumbar scoliosis and spinal deformity rather than measurement variability. Serial radiographic evaluations have documented thoracolumbar scoliosis with leftward convexity and loss of normal cervical lordosis, consistent with progressive spinal involvement. Upper segment to lower segment ratio (81.7 cm–59.5 cm) measured in April 2025 provides additional evidence of truncal height loss secondary to progressive spinal curvature abnormalities.

Musculoskeletal manifestations became increasingly prominent with advancing age. Joint contractures developed progressively affecting both large and small joints, with particular involvement of shoulders, fingers, and ankles. The characteristic tip-toe gait was observed, specifically affecting the right foot secondary to Achilles tendon contractures and rigid ankle joints. Exercise intolerance emerged with the patient reporting inability to walk extended distances and difficulty maintaining upright posture exceeding 1 minute duration.

Spinal abnormalities included thoracolumbar scoliosis with leftward convexity, loss of normal cervical lordosis, and presence of os odontoideum identified on cervical spine imaging. The patient experienced chronic generalized muscle discomfort, particularly affecting neck, shoulders, back, and limbs, contributing to functional limitations.

Ophthalmologic manifestations evolved during the follow-up period. Initial corneal topography performed at age 19 years (2015) revealed findings consistent with pellucid marginal degeneration in the right eye. Subsequent evaluations demonstrated progression to bilateral keratoconus, with the right eye more severely affected than the left. At age 26 years, bilateral glaucoma was diagnosed at a tertiary medical center, necessitating continuous ophthalmologic management.

The most recent comprehensive ophthalmologic assessment (November 2024, age 28 years) provided detailed characterization of the corneal abnormalities. Corneal topography demonstrated bilateral inferior steepening pattern characteristic of keratoconus, with simulated keratometry (Sim K) values of 47.67/43.65 diopters in the right eye and 46.55/43.83 diopters in the left eye. Zernike wavefront analysis confirmed significant higher-order aberrations including coma and trefoil components bilaterally. Notably, corneal pachymetry revealed markedly increased central corneal thickness measuring 693 μm in the right eye and 690 μm in the left eye, substantially exceeding normal population values which typically range from 520 to 560 μm. This paradoxical corneal thickening contrasts sharply with classical keratoconus presentations, where progressive stromal thinning typically results in central corneal thickness values below 500 μm in advanced cases.

This atypical presentation likely represents *ADAMTSL2*-related corneal stromal abnormality characterized by aberrant extracellular matrix deposition, rather than true ectatic keratoconus with stromal degradation. The topographic pattern of inferior steepening combined with paradoxical stromal thickening suggests a unique pathophysiology wherein dysregulated transforming growth factor-beta signaling leads to excessive extracellular matrix accumulation in the corneal stroma. The combination of bilateral glaucoma and keratoconus-like corneal ectasia with paradoxically increased corneal thickness has not been previously reported in geleophysic dysplasia and represents a novel *ADAMTSL2*-related ocular phenotype.

Cardiovascular evaluation revealed mild mitral valve prolapse with regurgitation on echocardiography, consistent with characteristic valvular involvement in geleophysic dysplasia. The patient experienced intermittent palpitations but remained hemodynamically stable throughout follow-up.

Gastrointestinal symptoms including frequent abdominal distension and bloody stool were observed in March 2025, prompting gastroenterology consultation. She experienced menstrual irregularities with initial amenorrhea lasting 5 months during adolescence, followed by recovery after medical treatment. Recent reduction in menstrual flow began at age 28 years and has persisted for more than 6 months.

### Physical examination findings

Physical examination revealed consciousness and alertness with distinctive skeletal dysplasia features. Anthropometric measurements included 53.8 cm head circumference and 132.5 cm arm span, both proportionally reduced relative to height, confirming severe short stature.

Craniofacial examination demonstrated bilateral ptosis with status post surgical correction, round face with full cheeks, short nose with anteverted nares, thickened nasal alae, and mild prognathism. These features contribute to the characteristic facial appearance observed in geleophysic dysplasia. Ophthalmologic assessment revealed conjunctival injection and abnormal temporomandibular joint movement with localized pain (left greater than right), confirming bilateral glaucoma.

**FIGURE 2:**
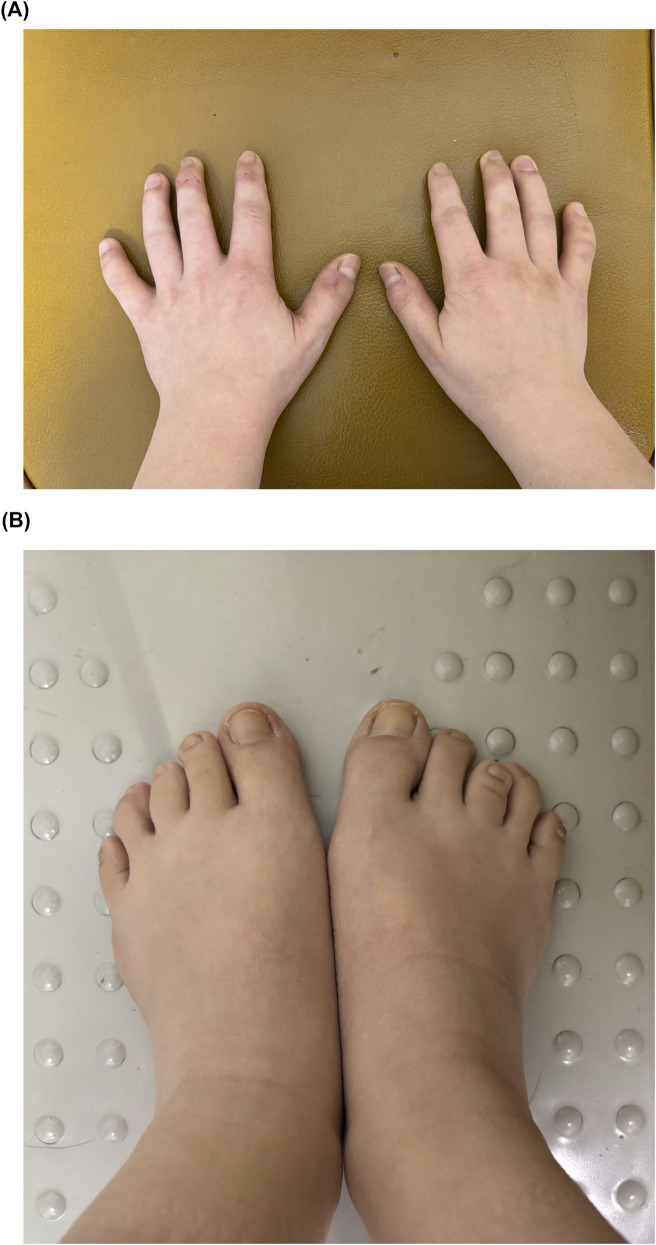
Clinical photographs of the 29-year-old patient. Panel **(A)**: Hands showing brachydactyly with severe limitation in finger extension, joint contractures with periarticular swelling at metacarpophalangeal and interphalangeal joints. Panel **(B)**: Feet showing proportionally small size with rigid ankle joints contributing to characteristic tip-toe gait.

Musculoskeletal examination revealed extensive joint contractures with severely limited range of motion affecting all small and large joints in flexion and extension. The patient could not make a full fist or fully extend both arms. Brachydactyly was present in both hands and feet with proportionally small distal extremities. Joint contractures with periarticular soft tissue swelling were particularly evident at metacarpophalangeal and distal interphalangeal joints (Figures 2A,B).

Spinal examination confirmed lordosis with limited range of motion and visible scoliosis. The right scapula was positioned higher than left, and the patient displayed abnormal gait patterns with persistent tip-toeing, particularly affecting the right foot. Cardiovascular examination revealed regular heart rhythm at 78 beats per minute with grade 1 to 2 out of 6 systolic murmur consistent with mitral regurgitation.

### Diagnostic investigations

Comprehensive metabolic screening was performed to exclude other skeletal dysplasias and metabolic disorders. Mucopolysaccharidosis was ruled out based on normal lysosomal enzyme activities including arylsulfatase B (26.3 μmol per gram protein per hour) and galactose-6-sulfate sulfatase (8.2 μmol per gram protein per hour). Additional lysosomal enzyme testing showed normal alpha-mannosidase activity (512.1 μmol per gram protein per hour), excluding alpha-mannosidosis.

Endocrine evaluation revealed normal thyroid function (thyroid-stimulating hormone 1.97 micro-international units per milliliter), appropriate reproductive hormone levels, and normal growth hormone axis function. Rheumatologic workup excluded rheumatoid arthritis with negative rheumatoid factor and negative HLA-B27.

Cardiovascular assessment included echocardiography confirming mild mitral valve prolapse with regurgitation, and electrocardiography revealing normal cardiac rhythm and conduction. Radiological investigations documented progressive skeletal abnormalities including thoracolumbar scoliosis, cervical spine straightening with os odontoideum, and characteristic hand bone changes.

**FIGURE 3:**
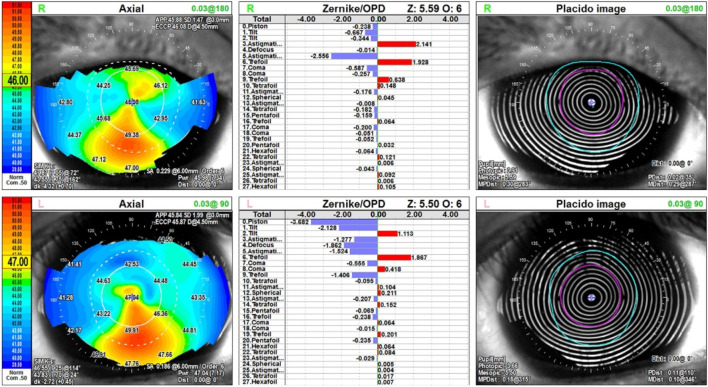
Corneal topography (NIDEK OPD-Scan III) demonstrating bilateral keratoconus with atypical features. Upper panels: Right eye showing inferior corneal steepening with simulated keratometry values of 47.67/43.65 diopters. Lower panels: Left eye showing similar inferior steepening pattern with simulated keratometry values of 46.55/43.83 diopters. Zernike wavefront analysis (middle columns) demonstrates elevated higher-order aberrations bilaterally. Placido disc images (right columns) confirm irregular corneal surface topography. Despite the keratoconus pattern, central corneal thickness was paradoxically increased (693 μm right eye, 690 μm left eye) rather than decreased, representing a novel *ADAMTSL2*-related corneal phenotype.

Comprehensive ophthalmologic evaluation included multiple imaging modalities to characterize the ocular manifestations. Corneal topography (NIDEK OPD-Scan III, November 2024) demonstrated bilateral keratoconus with characteristic inferior steepening pattern (Figure 3). Simulated keratometry values were 47.67/43.65 diopters at 72° (right eye) and 46.55/43.83 diopters at 114° (left eye). Zernike polynomial analysis revealed elevated higher-order aberrations, particularly astigmatism (coefficient −2.556 right eye, −1.524 left eye) and trefoil components bilaterally, consistent with irregular corneal surface morphology.

Corneal pachymetry revealed markedly increased central corneal thickness of 693 μm (right eye) and 690 μm (left eye), representing keratoconus-like corneal ectasia with paradoxical corneal thickening rather than the stromal thinning characteristic of classical keratoconus. This finding suggests aberrant extracellular matrix deposition in corneal stroma related to *ADAMTSL2* dysfunction.

Glaucoma assessment included optical coherence tomography of the optic nerve head (Carl Zeiss Cirrus HD-OCT, March 2024), which revealed asymmetric findings. Average retinal nerve fiber layer thickness was 93 μm (right eye) and 78 μm (left eye), with retinal nerve fiber layer symmetry of 72 percent. Average cup-to-disc ratio was 0.31 (right eye) and 0.54 (left eye), demonstrating notable asymmetry suggestive of glaucomatous changes predominantly affecting the left eye. Applanation tonometry showed elevated intraocular pressure of 25.0 mmHg (right eye) and 23.5 mmHg (left eye). However, the markedly increased central corneal thickness necessitates cautious interpretation, as thick corneas may lead to intraocular pressure overestimation by applanation tonometry. Corrected intraocular pressure values were calculated as 18.7 mmHg (right eye) and 17.4 mmHg (left eye), though the applicability of standard correction algorithms in this unique pathophysiology remains uncertain.

Automated perimetry (Haag-Streit Octopus 300 March 2024) demonstrated preserved visual fields with mean deviation of 0.6 dB and loss variance of 1.9, indicating no significant functional loss at present despite structural changes observed on optical coherence tomography. This dissociation between structural and functional findings warrants continued surveillance.

### Genetic testing results

Chromosomal analysis revealed normal 46,XX female karyotype. Initial microarray comparative genomic hybridization analysis did not detect specific pathogenic findings. Whole-exome sequencing performed in October 2023 identified compound heterozygous *ADAMTSL2* mutations (Table 1). The first mutation, c.286C>T, results in amino acid change from arginine to tryptophan at position 96 (p.Arg96Trp). In silico analysis predicts this variant to be deleterious (SIFT score 0.001; PolyPhen-2 score 0.998, probably damaging; MutationTaster disease-causing; CADD score 28.5). The arginine at position 96 is highly conserved across vertebrate species (PhyloP score 5.89; GERP++ score 5.45). The second mutation, c.454_459del, causes in-frame deletion of cysteine 152 and threonine 153 (p.Cys152_Thr153del). This deletion removes a cysteine residue that may be critical for disulfide bond formation and proper protein folding, and the affected region demonstrates high cross-species conservation. MutationTaster predicts this variant to be disease-causing.

**TABLE 1:** Whole-exome sequencing results showing compound heterozygous *ADAMTSL2* variants identified in the patient. Gene: *ADAMTSL2* (chromosome 9q34.2, autosomal recessive inheritance). Mutation 1: c.286C>T, p.Arg96Trp, missense variant, heterozygous, classified as variant of uncertain significance (VUS); *in silico* predictions (SIFT deleterious, PolyPhen-2 probably damaging, MutationTaster disease-causing, CADD 28.5) and high evolutionary conservation (PhyloP 5.89, GERP++ 5.45) support potential pathogenicity. Mutation 2: c.454_459del, p.Cys152_Thr153del, in-frame deletion removing a cysteine residue critical for disulfide bond formation, heterozygous, classified as VUS; MutationTaster predicts disease-causing. Combined status: compound heterozygous, likely disease-causing based on clinical correlation with geleophysic dysplasia phenotype. Quality metrics confirmed adequate coverage depth (>20x for target regions) and variant calling quality passing filters.

Patient information	
Parameter	Details
Age at testing	27 years
Sex	Female
Testing date	16 October 2023
Report date	13 January 2024
Gene analysis	
Gene	*ADAMTSL2*
Chromosome location	9q34.2
Inheritance pattern	Autosomal recessive
Mutation 1	
Nucleotide change	c.286C>T
Amino acid change	p.Arg96Trp (p.R96W)
Mutation type	Missense
Allele status	Heterozygous
Clinical significance	Variant of uncertain significance (VUS)
SIFT prediction	Deleterious (score 0.001)
PolyPhen-2 prediction	Probably damaging (score 0.998)
MutationTaster prediction	Disease-causing
CADD score	28.5
Conservation (PhyloP)	5.89 (highly conserved)
Conservation (GERP++)	5.45 (highly conserved)
gnomAD frequency	0.00001
Mutation 2	
Nucleotide change	c.454_459del
Amino acid change	p.Cys152_Thr153del
Mutation type	In-frame deletion
Allele status	Heterozygous
Clinical significance	Variant of uncertain significance (VUS)
MutationTaster prediction	Disease-causing
Conservation	Highly conserved region
Functional impact	Deletion of cysteine residue potentially disrupts disulfide bond formation
gnomAD frequency	Not reported (novel)
Combined status	
Zygosity	Compound heterozygous
Disease association	Geleophysic dysplasia (OMIM #231050)
Inheritance confirmed	Autosomal recessive
Clinical interpretation	Likely disease-causing based on clinical correlation
Quality metrics	
Coverage depth	>20x for target regions
Variant calling quality	PASS
Confirmation method	Sanger sequencing

Sanger sequencing validated presence of both compound heterozygous mutations, establishing the molecular diagnosis of geleophysic dysplasia (Table 2). Parental testing confirmed each parent carries one heterozygous mutation, consistent with autosomal recessive inheritance.

**TABLE 2:** Sanger sequencing confirmation results validating the compound heterozygous mutations and establishing family segregation pattern. Testing method: Sanger sequencing at MacKay Memorial Hospital (April 2025). Mutation 1 (c.286C>T, p.Arg96Trp, Exon 3): Patient heterozygous, paternal inheritance confirmed, maternal wild-type. Mutation 2 (c.454_459del, p.Cys152_Thr153del, Exon 4): Patient heterozygous, maternal inheritance confirmed, paternal wild-type. Inheritance pattern: autosomal recessive. Both variants classified as variants of uncertain significance based on ACMG criteria, though clinical correlation supports their pathogenic role in this patient.

Parameter	Mutation 1	Mutation 2
Sequencing information		
Testing method	Sanger sequencing	Sanger sequencing
Gene	*ADAMTSL2*	*ADAMTSL2*
Confirmation date	22 April 2025	22 April 2025
Laboratory	MacKay Memorial hospital	MacKay Memorial hospital
Mutation details		
Nucleotide change	c.286C>T	c.454_459del
Amino acid change	p.Arg96Trp	p.Cys152_Thr153del
Exon location	Exon 3	Exon 4
Mutation type	Missense variant	In-frame deletion
Inheritance analysis		
Patient status	Heterozygous	Heterozygous
Paternal inheritance	Confirmed carrier	Wild-type
Maternal inheritance	Wild-type	Confirmed carrier
Inheritance pattern	Autosomal recessive	Autosomal recessive
Quality assessment		
Sequence quality	High	High
Peak clarity	Clear heterozygous pattern	Clear deletion pattern
Confirmation status	Confirmed	Confirmed
Clinical interpretation		
ACMG classification	Variants of uncertain significance*	Variants of uncertain significance*
Disease association	Geleophysic dysplasia	Geleophysic dysplasia

### Treatment and management

The management strategy focuses on multidisciplinary care addressing progressive multisystem manifestations. Orthopedic consultation provided recommendations for joint contracture management and mobility preservation through physical therapy and adaptive equipment.

Ophthalmologic management encompasses both glaucoma control and keratoconus surveillance. Glaucoma management in this patient is complicated by the markedly increased central corneal thickness (693 μm right eye, 690 μm left eye), which significantly affects intraocular pressure measurement accuracy. Standard Goldmann applanation tonometry recorded elevated pressures of 25.0 mmHg (right eye) and 23.5 mmHg (left eye); however, thick corneas are known to cause overestimation of true intraocular pressure. Corrected intraocular pressure values were calculated as 18.7 mmHg (right eye) and 17.4 mmHg (left eye), falling within normal range. Given the uncertainty regarding the applicability of conventional correction algorithms in this novel pathophysiology, clinical decision-making relies primarily on structural and functional parameters rather than absolute pressure values.

Optical coherence tomography revealed asymmetric findings with average retinal nerve fiber layer thickness of 93 μm (right eye) and 78 μm (left eye), cup-to-disc ratios of 0.31 (right eye) and 0.54 (left eye), and retinal nerve fiber layer symmetry of 72 percent, suggesting early glaucomatous changes predominantly affecting the left eye. Automated perimetry demonstrated preserved visual fields with mean deviation of 0.6 dB, consistent with preperimetric glaucoma or glaucoma suspect status where structural damage precedes detectable functional loss.

Current glaucoma surveillance protocol includes intraocular pressure measurements every three to 4 months interpreted in context of central corneal thickness, annual optical coherence tomography to monitor retinal nerve fiber layer thickness trends, and annual automated perimetry to detect functional deterioration. Topical intraocular pressure-lowering therapy will be initiated if evidence of structural progression on optical coherence tomography or functional decline on perimetry emerges. Target intraocular pressure will be individualized based on the rate of retinal nerve fiber layer loss rather than predetermined numerical thresholds given the confounding effect of abnormal corneal thickness on pressure measurements.

For keratoconus management, the patient is currently corrected with spectacles (right eye: +3.25/-5.0 × 155°; left eye: +4.25/-2.75 × 15°) achieving best-corrected visual acuity of 0.8 (right eye) and 0.9 (left eye). Given the atypical corneal morphology with paradoxical stromal thickening rather than thinning, conventional keratoconus interventions require careful consideration. Corneal cross-linking, the standard treatment for progressive keratoconus aimed at strengthening weakened corneal stroma, may not be appropriate given the already thickened corneal stroma in this patient. Similarly, contact lens fitting has not been pursued given adequate visual function with spectacle correction and the unique corneal biomechanics.

The comprehensive ophthalmologic surveillance protocol includes serial corneal topography and pachymetry every six to 12 months to monitor keratoconus progression, regular intraocular pressure measurements with central corneal thickness correction, optical coherence tomography assessment of retinal nerve fiber layer and optic nerve head annually, and automated perimetry to detect functional deterioration. This multifaceted approach addresses both the corneal dystrophy and glaucoma manifestations unique to *ADAMTSL2*-related disease.

Cardiology follow-up includes annual echocardiographic surveillance given that cardiac valvular disease represents the primary determinant of prognosis in geleophysic dysplasia. The most recent echocardiography (April 2025) demonstrated normal left ventricular systolic function with ejection fraction of 70.7 percent, left ventricular end-diastolic diameter of 3.96 cm (normal range: 3.63–4.91 cm), and left ventricular end-systolic diameter of 2.39 cm (normal range: 2.17–3.29 cm). Mitral valve evaluation revealed mild prolapse of the slightly thickened anterior leaflet with mild regurgitation (Grade 1). Trivial tricuspid regurgitation (pressure gradient 18 mmHg) and trivial pulmonic regurgitation were noted. Electrocardiography showed normal sinus rhythm with normal conduction. Functionally, the patient is classified as New York Heart Association Class I, with exercise tolerance limited primarily by musculoskeletal manifestations rather than cardiac dysfunction. Serial echocardiographic assessments have demonstrated stable findings without significant valvular disease progression throughout the follow-up period, representing a favorable cardiac outcome compared to the high early mortality reported in geleophysic dysplasia.

The patient continues regular follow-up across multiple specialties, emphasizing quality of life maintenance while monitoring for disease progression and managing emerging complications.

## Discussion

### Clinical significance and disease progression

Geleophysic dysplasia represents one of the most severe skeletal dysplasia forms with considerable morbidity and mortality implications. The present case demonstrates characteristic progression from childhood presentation to adult survival, providing valuable insights into long-term natural evolution of this rare condition. The patient’s 25-year follow-up represents one of the longest documented cases in literature.

The patient’s clinical course aligns with established geleophysic dysplasia progression patterns described in landmark studies (Marzin et al., 2021). However, the patient’s survival to age 29 years represents favorable outcome, considering approximately 33 percent of individuals with geleophysic dysplasia die before age 5 years due to cardiac, airway, and pulmonary complications (Hubmacher et al., 2015).

### Novel ophthalmologic manifestations

In this patient, bilateral glaucoma identification at age 26 years is consistent with previously reported ocular manifestations in geleophysic dysplasia. Saricaoglu and colleagues reported bilateral angle-closure glaucoma requiring surgical intervention in a nine-year-old patient, and Zhang and colleagues documented characteristic ocular features predisposing to angle closure (Saricaoglu et al., 2013; Zhang et al., 2004). Our case contributes additional evidence supporting comprehensive ophthalmologic surveillance importance.

The identification of keratoconus-like corneal ectasia represents the primary novel finding in this case. Notably, central corneal thickness measured 690–693 μm, which markedly exceeds normal values. While bilateral glaucoma has been previously documented in geleophysic dysplasia, the paradoxical corneal thickening observed in our patient—contrasting sharply with the stromal thinning characteristic of classical keratoconus—has not been previously reported and likely reflects aberrant extracellular matrix deposition related to *ADAMTSL2* dysfunction. Classical keratoconus presents with progressive corneal thinning, typically with central corneal thickness values below 500 μm in advanced cases (Gomes et al., 2015). The paradoxical corneal thickening observed in our patient suggests a pathophysiology fundamentally distinct from classical keratoconus. In typical keratoconus, matrix metalloproteinase-mediated degradation of stromal collagen leads to progressive thinning and biomechanical weakening (Gomes et al., 2015). In contrast, the corneal findings in our patient likely represent aberrant extracellular matrix accumulation resulting from *ADAMTSL2* dysfunction. *ADAMTSL2* plays a critical role in fibrillin microfibril assembly and composition, and its loss of function may lead to excessive or disorganized deposition of extracellular matrix proteins including fibrillin-1, fibrillin-2, and associated glycoproteins in the corneal stroma (Le Goff et al., 2008; Camarena et al., 2024). This accumulation would explain the paradoxical increase in corneal thickness despite the presence of ectatic topographic features. Therefore, we propose designating this presentation as “keratoconus-like corneal ectasia” to accurately reflect the topographic similarities while acknowledging the distinct underlying pathophysiology. This *ADAMTSL2*-related corneal phenotype may represent a form of corneal stromal dystrophy characterized by matrix accumulation rather than true ectatic disease.

Several pathophysiological mechanisms may explain these ophthalmologic findings. Abnormal extracellular matrix deposition in ocular tissues, particularly in trabecular meshwork, Schlemm’s canal, and corneal stroma, may result from dysregulated transforming growth factor-beta signaling resulting from *ADAMTSL2* mutations. Studies have demonstrated that elevated transforming growth factor-beta levels in ocular tissues increase expression of extracellular matrix proteins such as fibronectin and collagen, resulting in increased tissue thickness and altered biomechanical properties (Wordinger et al., 2014; Fuchshofer and Tamm, 2012).

The characteristic geleophysic dysplasia craniofacial features including round face with full cheeks and potential orbital anatomical variations may contribute to anterior segment abnormalities predisposing to glaucoma development. Previous reports documented characteristic ocular features including shallow anterior chambers and thick peripheral iris, which are known risk factors for angle-closure glaucoma (Zhang et al., 2004).

These findings strongly support inclusion of comprehensive ophthalmologic surveillance in standard care protocols for *ADAMTSL2*-related geleophysic dysplasia. Such surveillance should include regular intraocular pressure measurements, gonioscopy to assess angle anatomy, optic nerve evaluation, and corneal pachymetry, ideally beginning in early childhood.

### Genetic correlations and molecular mechanisms

Molecular diagnosis in our case was established through whole-exome sequencing, revealing compound heterozygous *ADAMTSL2* mutations: c.286C>T (p.Arg96Trp) and c.454_459del (p.Cys152_Thr153del). The c.286C>T variant has been previously reported in ClinVar and classified as variant of uncertain significance, exhibiting extremely low population frequency (gnomAD: 0.00001). The c.454_459del variant has not been previously reported, suggesting it may be novel. Both variants remain classified as variants of uncertain significance (VUS) according to current ACMG criteria. However, the compound heterozygous state of these rare variants, combined with highly specific geleophysic dysplasia phenotype, supportive *in silico* predictions, high evolutionary conservation, and consistent family segregation data, provides strong circumstantial evidence suggesting these variants are likely disease-causing in this clinical context (Manickam et al., 2021). Functional studies would be required for definitive pathogenicity classification.

*ADAMTSL2* encodes a secreted glycoprotein essential for extracellular matrix organization and transforming growth factor-beta bioavailability regulation. Functional studies demonstrate that *ADAMTSL2* mutations lead to reduced secretion of mutated proteins, possibly due to protein misfolding (Batkovskyte et al., 2023). The protein interacts with latent transforming growth factor-beta-binding protein 1, and mutations result in dysregulated transforming growth factor-beta signaling, considered the underlying pathogenetic mechanism (Le Goff et al., 2008).

Through a recent study published in 2025, Morales and colleagues identified matrix metalloproteinase dysregulation in geleophysic dysplasia (Morales et al., 2025). The study demonstrated that fibroblasts from patients with *ADAMTSL2* variants exhibited increased cell migration associated with upregulation of matrix metalloproteinase-1 and matrix metalloproteinase-14. This finding represents the first potential disease-modifying therapeutic target beyond supportive care.

### Management considerations and therapeutic perspectives

Geleophysic dysplasia management requires comprehensive multidisciplinary approach addressing progressive multisystem manifestations. Following evidence-based guidelines, surveillance protocols should include annual cardiac evaluation with echocardiography, respiratory assessment for airway obstruction, and orthopedic monitoring for progressive joint contractures (Marzin et al., 2021).

The emergence of bilateral glaucoma and keratoconus-like corneal ectasia requires including comprehensive ophthalmologic surveillance in standard protocols. The discovery of matrix metalloproteinase dysregulation as therapeutic target offers hope for future disease-modifying treatments (Morales et al., 2025). Current management remains primarily supportive, focusing on preventing complications and maintaining quality of life.

### Limitations and future directions

Several limitations affect geleophysic dysplasia diagnosis, management, and research. Its extreme rarity creates substantial barriers to conducting controlled clinical trials and establishing evidence-based treatment protocols. The diagnostic complexity is exemplified by our case, where compound heterozygous variants were classified as variants of uncertain significance despite clinical correlation with geleophysic dysplasia.

The identification of novel phenotypic features, such as bilateral glaucoma and keratoconus-like corneal ectasia in the presented case, highlights evolving understanding of geleophysic dysplasia clinical spectrum. This necessitates continuous updates to diagnostic criteria and management protocols as new manifestations are recognized. Future research priorities should include functional validation of variants of uncertain significance, development of biomarkers for disease monitoring, and establishment of international registries.

## Conclusion

This case report presents comprehensive 25-year longitudinal follow-up of geleophysic dysplasia in a 29-year-old Taiwanese woman, representing one of the longest documented clinical courses in literature. The patient demonstrated multisystem manifestations typical of geleophysic dysplasia including severe short stature, extensive joint contractures, spinal abnormalities, and cardiac involvement.

Molecular diagnosis was supported by whole-exome sequencing identifying compound heterozygous *ADAMTSL2* variants: c.286C>T (p.Arg96Trp) and c.454_459del (p.Cys152_Thr153del). Although both variants are currently classified as VUS, the clinical phenotype, *in silico* predictions, evolutionary conservation, and family segregation data collectively suggest these variants are likely disease-causing in this patient. These findings follow autosomal recessive inheritance patterns and establish the genetic basis for this patient’s phenotype.

This case documents bilateral glaucoma, consistent with previously reported ocular manifestations, alongside keratoconus-like corneal ectasia with markedly increased central corneal thickness measuring 690–693 μm. The paradoxical corneal thickening represents a novel *ADAMTSL2*-related corneal phenotype not previously documented in geleophysic dysplasia. This corneal thickening contrasts with classical keratoconus presentations and likely reflects aberrant extracellular matrix deposition related to *ADAMTSL2* dysfunction. These findings indicate comprehensive ophthalmologic surveillance including corneal pachymetry should be incorporated into standard management protocols for patients with *ADAMTSL2* mutations.

The patient’s survival to adulthood with maintained intellectual function demonstrates variable severity within geleophysic dysplasia spectrum. This case report emphasizes the importance of multidisciplinary management approaches including orthopedic, cardiac, ophthalmologic, and genetic counseling services. Future studies should focus on establishing standardized surveillance protocols incorporating newly recognized manifestations while investigating potential therapeutic targets including recently identified matrix metalloproteinase dysregulation pathways.

## Data Availability

The original contributions presented in the study are included in the article/supplementary material, further inquiries can be directed to the corresponding authors.
